# Extension of the Life Span by Acarbose: Is It Mediated by the Gut Microbiota?

**DOI:** 10.14336/AD.2022.0117

**Published:** 2022-07-11

**Authors:** Baiyun Wu, Jiai Yan, Ju Yang, Yanping Xia, Dan Li, Feng Zhang, Hong Cao

**Affiliations:** ^1^Nutritional Department, Affiliated Hospital of Jiangnan University, Wuxi, China.; ^2^School of Medicine, Nantong University, Nantong, China.; ^3^Clinical Assessment Center of Functional Food, Affiliated Hospital of Jiangnan University, Wuxi, China.; ^4^Department of Endocrinology, Affiliated Hospital of Jiangnan University, Wuxi, China.

**Keywords:** acarbose, life span, gut microbiota

## Abstract

Acarbose can extend the life span of mice through a process involving the gut microbiota. Several factors affect the life span, including mitochondrial function, cellular senescence, telomere length, immune function, and expression of longevity-related genes. In this review, the effects of acarbose-regulated gut microbiota on the life span-influencing factors have been discussed. In addition, a novel theoretical basis for improving our understanding of the mechanisms by which acarbose extends the life span of mice has been suggested.

## 1. Introduction

Since ancient times, humans have shown considerable interest in extending their life spans, which has inspired many studies on the mechanism underlying the extension of the life span. Recent studies have demonstrated that the human life span is affected by inflammation, mitochondrial function, telomere length, and immune function [1-4]. Diabetes is a common disease among elderly adults, and its complications can lead to organ damage and shortening of the life span. Metformin is a first-line hypoglycemic drug that not only lowers blood glucose levels but also affects the human life span [5]. Metformin can reduce oxidative stress and ameliorate chronic inflammation, which may aid in extending the life span [6].

Acarbose, which was originally discovered to be produced by *Actinoplanes* sp. SE50/110, is used as a first-line treatment for diabetes [7]. As an inhibitor of α-glucosidase, acarbose delays carbohydrate digestion by competitively inhibiting α-glucosidases and pancreatic α-amylase, thereby reducing blood glucose levels in patients with type 2 diabetes mellitus (T2DM) [8]. In Asia, energy intake is mainly derived from carbohydrates, so acarbose is more commonly used in Asia. After acarbose treatment, the glycated hemoglobin level can be reduced by as much as 0.77%, which is predominantly noted in the Eastern Asian population and those consuming high-carbohydrate diets [9]. Acarbose supplementation can partially offset the age-related dysregulation of glucose and insulin [10,11]. Acarbose can attenuate many risk factors for cardiovascular diseases (CVDs) by reducing the incidence of postprandial hyperglycemia and ameliorating endothelial dysfunction. Acarbose can also improve blood glucose levels and risk factors associated with CVDs when combined with a moderate level of exercise [12]. Furthermore, acarbose-mediated reduction of post-prandial blood glucose levels can indirectly change glucose metabolism, thereby improving insulin sensitivity and affecting the life span [13].

Mechanistically, acarbose has been suggested to influence the human life span by modulating the gut microbiota [14]. Thus, acarbose treatment to maintain blood glucose levels may be a viable approach to maintain health and delay ageing [15].

## 2. Remodelling the gut microbiota can extend the lifespan

Increasing evidence suggests that the gut microbiota plays a vital role in the occurrence and development of T2DM [8]. Metformin promotes gut microbiota metabolites, such as short-chain fatty acids (SCFAs), which may play a crucial role in extending the life span of *Caenorhabditis elegans* [16]. Whether metformin affects the life span of worms depends on its sensitivity to *Escherichia coli*and glucose levels [17]. Similarly, acarbose has been reported to play a role in extending the life span of mice. Poor glucose regulation can accelerate the aging process. Therefore, acarbose can improve health and extend life span by regulating glucose metabolism. Resistant starch can resist degradation by digestive enzymes in the small intestine and reach the large intestine, suggesting that resistant starch modulates gut homeostasis, similar to acarbose [18]. Resistant starch can increase the abundance of SCFAs, especially butyric acid [19,20]. The effect of acarbose on longevity may be mediated by glucose physiology and microbial activity in the intestine. Acarbose can increase the amount of starch that enters the colon, thereby enriching carbohydrate-degrading bacteria and altering the gut microbiota and its fermentation products [21]. Hence, treatment with acarbose can increase the abundance of potential SCFA-producing bacteria [8,14].

Nutritionally, calorie restriction (CR) is considered an effective strategy for extending the life span [22]. Complex interactions between metabolic adaptations and immune and anti-inflammatory responses may contribute to the health-promoting and longevity effects of CR [23,24]. CR can considerably alter the gut microbiota of aging mice [25] and reduce age-related changes in the gut microbiota [26]. *Bifidobacterium* possesses immune-modulatory and anti-inflammatory effects; therefore, it is believed to be beneficial for colon health [27,28]. In calorie-restricted mice, the decline in the relative abundance of age-related bacteria, such as *Bifidobacterium* and *Akkermansia*, could be prevented [26]. Remodeling of the gut microbiota during CR contributes to improved insulin sensitivity and glucose tolerance and decreases fat gain and the formation of beige adipocytes, which in turn extends the life span [29]. Similar to the effect of CR on glucose metabolism and insulin activity in rodents and primates, acarbose supplementation can improve age-related glucose and insulin disorders [15]. The therapeutic effects of acarbose treatment are achieved by the selective inhibition of carbohydrate metabolism for calorie compensation while allowing carbohydrate consumption [30].

Recent studies on CR mimetic (CRM) compounds revealed that acarbose belongs to the upstream type of CRMs and regulates glucose metabolism [31]. CR diet induces homeostasis of the gut microbiota, which is potentially beneficial for health promotion and indicates a close relationship between regulation of the gut microbiota and healthy aging [25,31]. Although CR can extend the life span, implementing long-term CR is quite challenging. Therefore, the use of a CRM compound, such as acarbose, instead of a CR diet, would be more practical and acceptable for patients with diabetes [30,32].

Although direct evidence of the roles of gut microbiota in extending the life span by using acarbose is scarce, the following aspects may be helpful in understanding the underlying mechanism of how acarbose affects the life span as mediated by the gut microbiota. First, how acarbose influences the composition and function of the gut microbiota is discussed. Second, how changes in the gut microbiota influence life span are discussed. Third, the potential gut microbiota-mediated mechanism for the extension of life span by using acarbose is elaborated.

## 3. Acarbose influence the composition and functions of the gut microbiota

The effect of acarbose on intestinal fermentation products may influence its overall effect on host physiology. SCFAs are the main products of starch fermentation of intestinal bacteria, which are beneficial to health, and a positive correlation has been reported between SCFA levels in mice feces and their survival rates [14]. Acarbose could influence both the composition of the bacterial community and SCFA levels in mice feces. Changes in the butyric and acetic acid levels after acarbose treatment vary in different populations from different countries. The acetic acid and butyric acid levels were found to increase after acarbose treatment in most populations in Germany and the United States [33-35], but no significant difference was observed in the butyric acid level after acarbose treatment in diabetic populations in China [36]. In nondiabetic people in the United States, the butyric acid levels increased, whereas the acetic acid levels did not significantly change after acarbose treatment [37]. In feces of centenarians in China, seven characteristic compounds (i.e., total SCFA; manganese; cobalt; and acetic, propionic, butyric, and valeric acids) were identified. This metabolic pattern, particularly the increase in the levels of total bile acids and SCFAs, may have an important and positive effect on longevity [38]. In another study of centenarians in Sardinia, their intestinal microbiota showed potential health promoting characteristics, which was related to the high capacity of glycolysis and SCFA production, leading to the extension of lifespan[39]. For instance, in mice fed with acarbose, the abundance of the SCFA-producing bacteria *Lactobacillus* increased [40]. Besides their anti-inflammatory effects, SCFAs can also regulate immunity [41]. In addition to well-known SCFAs, such as acetic, butyric, and propionic acids, other SCFAs have the ability to extend the life span. For example, formate can reduce the generation of reactive oxygen species (ROS), which can also extend the life span. Thus, acarbose affects longevity by influencing the abundance of SCFAs [42].

Remodeling the gut microbiota can also extend the life span, and acarbose is known to regulate the gut microbiota. However, to understand the role of gut microbiota in the extension of the life span by acarbose, it is important to investigate which factors influence the life span based on the gut microbiota and how the gut microbiota affect these influencing factors.

## 4. Factors that influence lifespan based on the gut microbiota

Aging features include mitochondrial dysfunction, cellular senescence, altered cell-to-cell communication, and loss of protein stabilization. Dysfunctional mitochondria in T cells have been reported to accelerate senescence [43]. With age, cellular stress and damage increase, and correspondingly, the levels of ROS increase in an attempt to increase the chances of survival. Beyond a certain threshold, these changes eventually aggravate age-associated damage [44]. Naturally, telomeres in somatic cells gradually shorten with age, ultimately leading to cellular senescence. Human cells expressing telomerase were shown to regulate telomere length. An increase in telomerase activity results in increased telomere length [45,46]. Immune responses, mitochondrial function, senescence-associated gene expression, and telomeres are the most important factors affecting the life span.

### 4.1 Immune responses

The innate immune system in mammals induces inflammatory responses, and age-related changes in inflammatory responses are considered to cause aging [47]. The levels of pro-inflammatory factors in the serum of the elderly are higher than those in younger people. The increase in the levels of pro-inflammatory factors is indicative of aging. Furthermore, changes in the activity of the innate immune system are associated with age-related diseases. Studies on *C. elegans* showed that the innate immune function is regulated by transforming growth factor-β (TGF-β), p38 mitogen-activated protein kinases, and the DAF-2 insulin pathway [48]. Dysregulated signaling in the TGF-β pathway plays a major role in inflammation, fibrogenesis, and immunomodulation [49]. DAF-2 plays an important role in the regulation of the life span partly by controlling immune system-related gene expression [50]. A sustained inflammatory response can damage the innate immune system, activate stress responses, and induce the impairment of cells and molecules [51]. Lower levels of interleukin (IL)-6 were detected in centenarians than older person [47,52]. The pathways of phosphatidylinositol signal transduction, sphingomyelin biosynthesis, and biosynthesis of various *N*-polysaccharides in the gut microbiota were higher in centenarians, indicating healthy immune functions and a balanced intestinal environment [53].

Accumulating evidence has shown that dysbiosis of the gut microbiota can cause inflammation and that probiotics can regulate the gut microbiota and reduce inflammation. The diversity of the gut microbiota decreases with age. Age-related gut microbiota disorder triggers the innate immune response and low-grade inflammation. This ultimately leads to intestinal dysplasia, which in turn leads to epithelial dysfunction, making the host susceptible to unhealthy aging, infection, and increased mortality [54]. The association between inflammation and the gut microbiota suggests the role of inflammation in the extension of the life span, which is at least partially modulated by the gut microbiota [55]. Intestinal alkaline phosphatase (IAP) is an anti-inflammatory enzyme that helps in clearing many bacteria-derived pro-inflammatory factors, such as LPS. The lack of IAP can promote aging-related inflammation in mice and shorten their life span; on the contrary, IAP supplementation can reduce intestinal permeability, maintain the steady state of the gut microbiota, and inhibit aging-related inflammation, thereby significantly extending the life span [56]. Dietary inulin can increase the levels of beneficial SCFA-producing bacteria, such as *Lactobacillus*, and inhibit the expression of inflammatory genes from extending the life span [57]. Probiotic-4, a probiotic formula consisting of *Bifidobacterium lactis, Lactobacillus casei, Bifidobacterium bifidum*, and *Lactobacillus acidophilus*, can decrease the expression level of IL-6 [58]. Therefore, the gut microbiota can affect longevity by influencing inflammation and immunity.

### 4.2 Mitochondrial function

Decreased mitochondrial function plays an important role in promoting aging in humans. Mitochondria may affect the life span mainly through cell aging, chronic inflammation, and stem cell dysfunction [59]. Aging is usually accompanied by reduced mitochondrial-related metabolic activity [44,59,60]. For instance, mutations in mice mitochondrial DNA polymerase lead to mitochondrial DNA deletion, which accelerates aging [61]. During mitochondrial respiration, the generation of ROS and free radicals, such as hydrogen peroxide, result in cumulative damage to DNA, proteins, and lipids, eventually leading to the loss of tissue function and ultimately, death [62]. This could be one of the mechanisms by which mitochondrial dysfunction affects longevity. Recently, it has been reported that the gene expression levels of mitochondrial superoxide dismutase 2 and fission 1 were decreased in aged mutant mice, suggesting that the shorter life span and poor physical condition of this mouse result from mitochondrial dysfunction [63]. Acetic acid and butyric acid produced by the gut microbiota via fermentation have protective effects on oxidative and mitochondrial stress. Thus, they can inhibit Streptozotocinn(STZ)-induced cell apoptosis, mitochondrial dysfunction, and excessive ROS generation [64]. This could further help in understanding the interactions between the gut microbiota and mitochondrial functions that affect the life span.

### 4.3 Telomeres

One of the main manifestations of aging is telomere shortening [65]. The length of telomeres decreases during a human’s lifetime, limiting cell proliferation, damaging cells, and ultimately shortening the life span. The length of telomeres determines the life span to some extent. Mutations in the gene encoding telomerase can lead to increased telomere shortening, which reduces the regenerative functions of stem cells [66] and activates the p53 pathway [67]. Telomere length and the telomere shortening rate (TSR) are recognized indicators of aging in population studies. Aging is always accompanied by low-grade systemic inflammation, which may be a result of the progression of age and age-related diseases, leading to telomere shortening [68]. SCFAs can regulate the immune response and show anti-inflammatory effects, which can result in the extension of telomere length [69].

### 4.4 Senescence-associated gene expression

Sirtuin (SIRT) is often referred to as the “longevity gene” because it plays an important role in protein and DNA repairs. SIRT1 and SIRT6 are promising regulators of longevity [70] that belong to the histone deacetylase sirtuin family [71]. Dysbiosis of the gut microbiota can promote neuroinflammation and neurodegeneration. In recent years, there has been increasing evidence that sirtuins play a role in obesity, diabetes, and various age-related neurodegenerative diseases. Although few direct studies have proved that the gut microbiota can influence the longevity gene, including genes belonging to the sirtuin family, metabolites derived from the gut microbiota affect the life span by affecting the longevity gene. SCFAs can promote gut-brain activation by regulating the gut microbiota. SIRT1 plays an important role in maintaining intestinal tissue homeostasis by regulating the gut microbiota1 plays an important role in maintaining intestinal tissue homeostasis by regulating the gut microbiota [72,73]. The butyrate levels in the intestine are positively correlated with the levels of life-prolonging hormone fibroblast growth factor 21, which participates in SIRT1 activation [74]. *L. mali* (APS1) can increase butyrate levels, activate glucagon like peptide-1(GLP-1) receptors, and enhance the expression of SIRT1 while inhibiting oxidative stress by activating SIRT1 [75]. The increase in serum oxidative status and the subsequent decrease in SIRT6 expression may be caused by the decreased population of *Firmicutes/Bacteroidetes* and the abundance of *Bifidobacterium*, suggesting that regulating the expression of the longevity gene by ameliorating gut dysbiosis may be beneficial to life span extension [76].

## 5. Putative gut microbiota-mediated mechanisms for the extension of the life span by using acarbose

The gut microbiota can regulate the life span by affecting inflammation. However, few studies have assessed the effect of the gut microbiota on oxidative stress or telomere length, and the underlying mechanisms remain unclear. In contrast, acarbose plays a significant role in regulating the gut microbiota, so how does acarbose regulate gut microbiota to affect the life span?

### 5.1 Anti-inflammatory response

In addition to its anti-hyperglycemic effect, acarbose exerts an anti-inflammatory effect [77]. Increased inflammatory activity is accompanied by brain aging, but acarbose can delay aging in mice by inhibiting the activation of the hypothalamic nuclear factor kappa B (NF-κB) inflammatory pathway [78]. Acarbose has been shown to inhibit interferon-γ inducible protein-10, monocyte chemoattractant protein-1, macrophage-derived chemokine, and TNF-α activation and to downregulate NF-κB-P65 activity in human monocytic THP-1 cells [77]. Probiotics can attenuate the expression of IL-6, which is negatively associated with longevity [52,58]. Furthermore, the levels of IL-6 in patients with diabetes treated with acarbose are also significantly reduced [79]. Adipose tissue inflammation plays an important role in senile diseases, and acarbose is presumed to extend the life span by slowing or preventing adipose tissue inflammation [80]. Acarbose may reduce the expression levels of inflammatory factors by increasing the abundance of beneficial bacteria. For example, acarbose treatment significantly increases the relative abundance of *Ruminococcus* and *Bifidobacterium. Ruminococcus* produces acetic and propionic acids, both of which can improve metabolic abnormalities and intestinal inflammation. Many species of *Bifidobacterium* and *Lactobacillus* are recognized as SCFA-producing bacteria that exert anti-inflammatory effects [81]. These findings provide strong evidence for the immunosuppressive effect and anti-inflammatory potentials of acarbose.

### 5.2 Reduced generation of mitochondrial ROS

Hyperglycemia and even a short-term increase in blood glucose levels activate vascular cells and cause endothelial dysfunction. These toxic effects may be a result of the generation of ROS in the vascular systems in diabetic and hyperglycemic states [82,83]. Mitochondrial aconitase is a sensitive marker of oxidative stress in aging-related diseases, and its diminished activity can serve as an indicator of ROS generation in mitochondria [84,85]. The activities of mitochondrial aconitase decreased in sucrose-fed obese Zucker rats with insulin resistance and hyperglycemia, suggesting that mitochondria generate ROS during dysfunctional glucose metabolism. Treatment with acarbose can largely prevent the reduced activity of mitochondrial aconitase, the increase in oxidative stress, and vascular dysfunction caused by hyperglycemia [86].

When the fecal microbiota of patients with depression was transplanted into germ-free rats, mitochondrial damage was observed in intestinal epithelial cells, indicating that intestinal microbial disorders result in mitochondrial damage [87]. As mentioned previously, SCFAs, such as acetic and butyric acids, inhibit mitochondrial dysfunction and ROS overproduction. Furthermore, mopping up ROS or improving mitochondrial dysfunction can delay aging [55]. Based on this, we can speculate that acarbose further extends the life span by increasing the abundance of SCFAs.

### 5.3 Influence on telomere length

Some dietary components can affect the length of telomeres and lead to aging. For example, excessive sugar consumption can shorten telomeres, whereas increased consumption of plant-based foods rich in antioxidants can maintain telomere length [88]. Because acarbose decreases carbohydrate absorption, it can theoretically extend the length of telomeres. However, acarbose treatment was shown to increase TSR, which accelerates the biological aging of patients. In addition to delaying sucrose and starch digestion to reduce blood glucose levels, acarbose may also disrupt gastrointestinal transport, leading to metabolic disorders that accelerate telomere shortening [89]. Although acarbose was found to increase TSR, in later studies by the same group, this effect was not discovered in all populations but only in certain patients with diabetes. Acarbose was found to show different effects on telomeres in different populations with severe insulin-resistant diabetes (SIRD) and non-SIRD [90].

Acarbose has been widely regarded as increasing the abundance of SCFAs, especially acetic and butyric acids. Using yeast as a model organism, Romano et al. found that acetic acid had the capability of extending the length of telomeres [69]. Butyric acid consumption led to an earlier peak in plasma triglyceride levels and increased plasma total cholesterol levels, which might be responsible for shortening telomere length in postmenopausal women [91]. Treatment with acarbose had various effects on the levels of acetic and butyric acids in different mice strains [14,92,93]. Furthermore, as mentioned previously, the levels of butyric and acetic acids varied in different populations after acarbose treatment, and the levels of butyric acid increased after acarbose treatment in most populations, which may partly explain why treatment with acarbose resulted in shortening of telomere length.

It has been found that the mechanisms by which acarbose affects telomeres differ between patients with and without SIRD [90]. In this article, Huang et al. mentioned that a study they cited had reported that the abundance of *Bifidobacterium* decreased after acarbose administration and suggested that the reduced abundance of *Bifidobacterium*, which aggravated inflammation, and the gut microbiota dysbiosis in the SIRD group are characterized by insulin resistance after acarbose treatment. They then presumed that the disordered gut microbiota plays an important role in telomere attrition in patients with SIRD receiving acarbose treatment. However, the study they cited actually showed that the abundance of *Bifidobacterium* increased after acarbose treatment, which could not account for the role of gut bacteria in shortening telomere length [40]. They did not further examine the levels of butyric and acetic acids. To further demonstrate the role of the gut microbiota in the regulation of telomere length, it is necessary to study the abundance of butyric and acetic acid producers and the levels of SCFA in various populations. In addition, considering that telomere length is not an independent factor affecting the life span, whether acarbose affects life span extension by mainly regulating the inflammatory level and mitochondrial function based on the gut microbiota needs further investigation.

### 5.4 Influence on longevity genes

Similar to acarbose, galacto-oligosaccharide (GOS) cannot be absorbed in the small intestine, so more carbohydrates reach the large intestine. GOS regulates the gut microbiota and its metabolites through the liver-gut axis. GOS fermentation significantly increases butyrate acid levels, which can activate the hepatic adenosine 5'-monophosphate (AMP)-activated protein kinase (AMPK)/SIRT1 signaling pathway, increase SIRT1 expression, restore hepatic antioxidant activity, and alleviate aging [94]. However, there is no direct evidence that acarbose affects the SIRT6 longevity gene.

Acarbose may extend the life span by regulating the gut microbiota and thus, attenuating the inflammatory reaction, which is the risk of the disorder of the mitochondrial function and the telomere attrition. However, there is no direct evidence that acarbose can extend the life span by regulating aging-related genes and telomere length, which is a subject that should be further examined.

**Figure 1.**
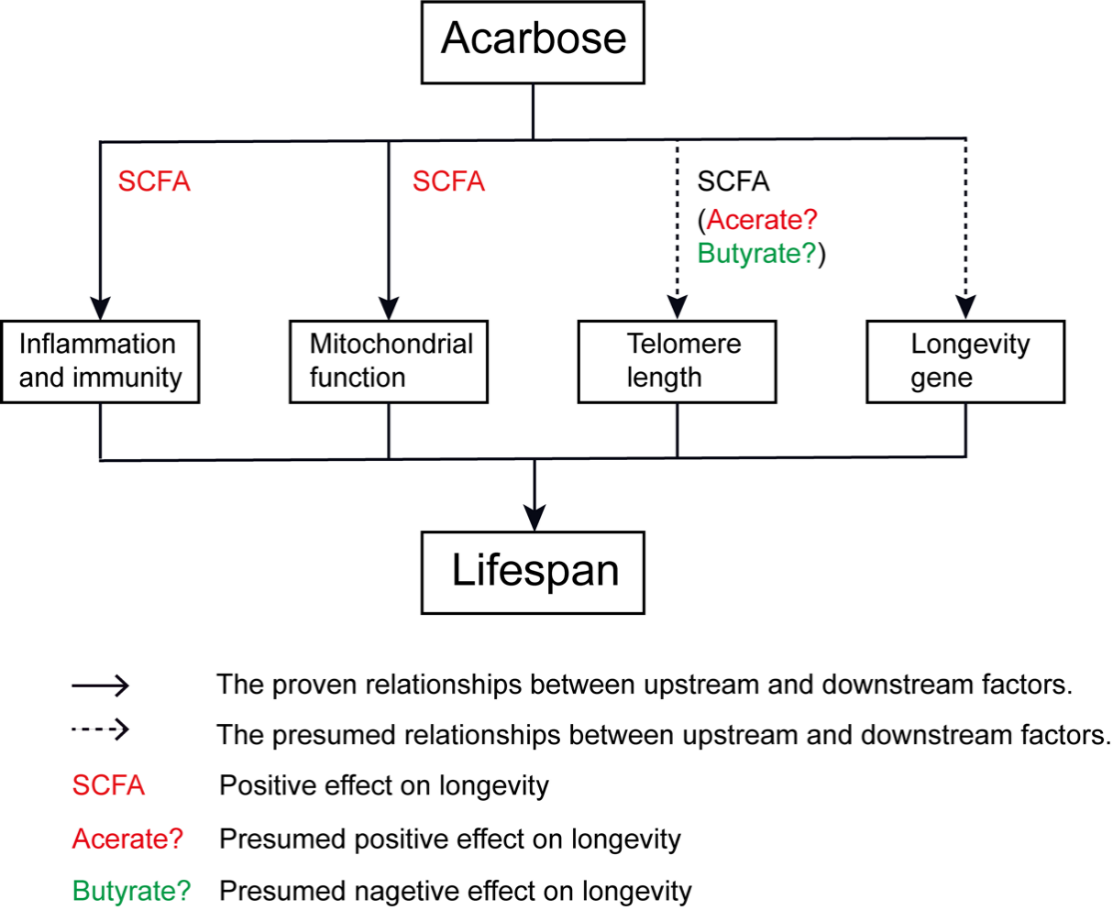
Potential mechanisms by which acarbose extends life span mediated by the gut microbiota.

## 6. Conclusion

The existing literature provides evidence that acarbose can affect the life span. This review linked inflammation, mitochondria, and telomeres with the gut microbiota, illustrating individual mechanisms involved in acarbose-associated life span extension. Acarbose improves the immune system, inflammatory response, and mitochondrial function by affecting the gut microbiota. Acarbose supplementation is a cost-effective method for delaying aging given its potential health-restorative effects and limited side effects. This offers hope for analyzing the use of acarbose to improve health and reduce the risk of age-related diseases.

Additional experiments should be undertaken to verify our speculations; for instance, which bacteria affect the length of telomeres and mitochondrial function after acarbose intervention needs to be studied. The role of acarbose in affecting telomere length by regulating the gut microbiota should be investigated with a more rigorous scientific approach. The present review describes several mechanisms by which acarbose affects the life span through the gut microbiota by considering different viewpoints and provides a new theoretical basis for the mechanism of acarbose-extended life spans. Hitherto, to the best of our knowledge, no other reviews have explained the mechanisms underlying life span extension by acarbose based on the perspective of gut microbiota. In general, many factors that affect the life span and mechanisms of acarbose that can help extend the life span of humans remain to be studied.
